# Microevolution during an Anthrax Outbreak Leading to Clonal Heterogeneity and Penicillin Resistance

**DOI:** 10.1371/journal.pone.0089112

**Published:** 2014-02-13

**Authors:** Joakim Ågren, Maria Finn, Björn Bengtsson, Bo Segerman

**Affiliations:** 1 Department of Bacteriology, National Veterinary Institute (SVA), Uppsala, Sweden; 2 Department of Animal Health and Antimicrobial Strategies, National Veterinary Institute (SVA), Uppsala, Sweden; 3 Department of Biomedical Sciences and Veterinary Public Health, Swedish University of Agricultural Sciences (SLU), Uppsala, Sweden; St. Petersburg Pasteur Institute, Russian Federation

## Abstract

Anthrax is a bacterial disease primarily affecting grazing animals but it can also cause severe disease in humans. We have used genomic epidemiology to study microevolution of the bacterium in a confined outbreak in cattle which involved emergence of an antibiotic-resistant phenotype. At the time of death, the animals contained a heterogeneous population of Single Nucleotide Variants (SNVs), some being clonal but most being subclonal. We found that independent isolates from the same carcass had similar levels of SNV differences as isolates from different animals. Furthermore the relative levels of subclonal populations were different in different locations in the same carcass. The heterogeneity appeared to be derived in part from heterogeneity in the infectious dose. The resistance phenotype was linked to clonal mutations in an anti-sigma factor gene and in one case was preceded by an acquisition of a hypermutator phenotype. In another animal, small subclonal populations were observed with counteracting mutations that had turned off the resistance genes. In summary, this study shows the importance of accounting for both acquired and inherited heterogeneity when doing high-resolution infection tracing and when estimating the risks associated with penicillin treatment.

## Introduction

As genomic sequencing has become cheaper, faster, and more accessible, new application areas have opened. In the emerging field of genomic epidemiology, ultra high-resolution Next Generation Sequencing (NGS) is used to distinguish different isolates from the same infectious disease outbreak [1]. The power of genomic sequencing to analyze infectious disease outbreaks has been explored for several pathogens including *Bacillus anthracis* [2] and in particular for *Mycobacterium tuberculosis*, it is now even possible to resolve individual transmission events [3], [4], [5], [6], [7], [8], [9], [10]. This type of high-resolution analysis of outbreak isolates gives insights into how mutation rates and microevolution reshape the genome during an infection. *M. tuberculosis* is an extremely slow growing bacterium with a long course of infection. Here we show how microevolution in a fast growing bacterium, *B. anthracis*, from several parallel *in vivo* cases within the same confined outbreak, independently gave rise to antibiotic resistance.

*B. anthracis* is a gram positive, spore-forming bacterium that is the causative agent of anthrax. This aggressive disease primarily affects grazing animals but many mammals are more or less susceptible. In humans, the most common form is a milder cutaneous infection causing black necrotic ulcers (hence the name anthrax which is Greek for coal). However, inhalation of spores can cause a more aggressive pulmonary anthrax form that is associated with high mortality rates [11]. *B. anthracis* is therefore classified as a risk-group 3 organism. Ingestion of spores can also cause a severe gastrointestinal form of anthrax. A new form has also been described referred to as injectional anthrax [12].

Anthrax spores are very inert. In their natural life cycle they can lie dormant in the ground for decades [13], until drought followed by heavy rains or other disturbances brings the spores to the surface thereby putting grazing animals at risk of infection. Spores from infected cattle carcasses buried several decades ago can put living animals at risk, especially since the exact location of these cattle graves is seldom known. An anthrax outbreak often starts from a single source of contamination, rapidly spreads, and then dies out.

*B. anthracis* is susceptible to beta-lactam antibiotics but resistance can occur, albeit in rare cases [14] and the risk for encountering resistant strains is still considered very low [15]. However, some studies suggest that up to 11% of the isolates may be resistant to beta-lactams [16] but as of 2004, only five cases of resistant isolates from fresh human or animal samples have been reported [17]. Therefore beta-lactams are still among the antibiotics recommended by WHO and CDC for post-exposure prophylaxis and for treatment of anthrax, albeit susceptibility testing is recommended [18], [19].

In Sweden, anthrax outbreaks were frequent in cattle during the first half of the 20th century. The disease then declined and only sporadic cases have been reported. In July-August 2011, an outbreak in cattle occurred in a Swedish nature reserve. The nature reserve contained grazing cattle over approximately one square km of seasonally flooded land. During the spring, several of the ditches had been dredged. At the end of July, several cows abruptly died of anthrax. In all, 24 animals died and 3 fetuses were aborted over a period of 4 weeks. Old archive documents showed that an anthrax burial site from the mid-1940s was located in the area although the exact position was not specified. Thus, it seemed likely that the grave had been disturbed which thereby exposed the cows to dormant anthrax spores. After the anthrax diagnosis had been confirmed, the remaining animals were treated with penicillin, pending vaccination, which halted the disease progression. However, a few animals also died during and after the treatment period and strains isolated from these animals were shown to be penicillin resistant.

We here show how microevolution in several parallel *in vivo* cases within the same confined outbreak, independently gave rise to antibiotic resistance in *B. anthracis*.

## Results

### Genomic Epidemiology Analysis of the Outbreak Strains

*B. anthracis* is a monophyletic species with an extraordinarily small genetic variability [20]. The complete genome sequence of the first diagnosed case (Cow1) was determined using a combination of 454, Illumina, and Sanger reads. The chromosome comprised 5.2 Mb and when looking at the 13 canonical SNP (canSNP) positions identified by Van Ert et al. the strain belongs to the B.Br.001/002 lineage [21]. Only ten ribosomal RNA operons were present as compared to the eleven which are most often found in this species. However, variation in the number of ribosomal operons has been observed previously [22], [23]. The nucleotide differences in all remaining isolates were determined using Cow1 as a reference sequence.

**Figure 1:**
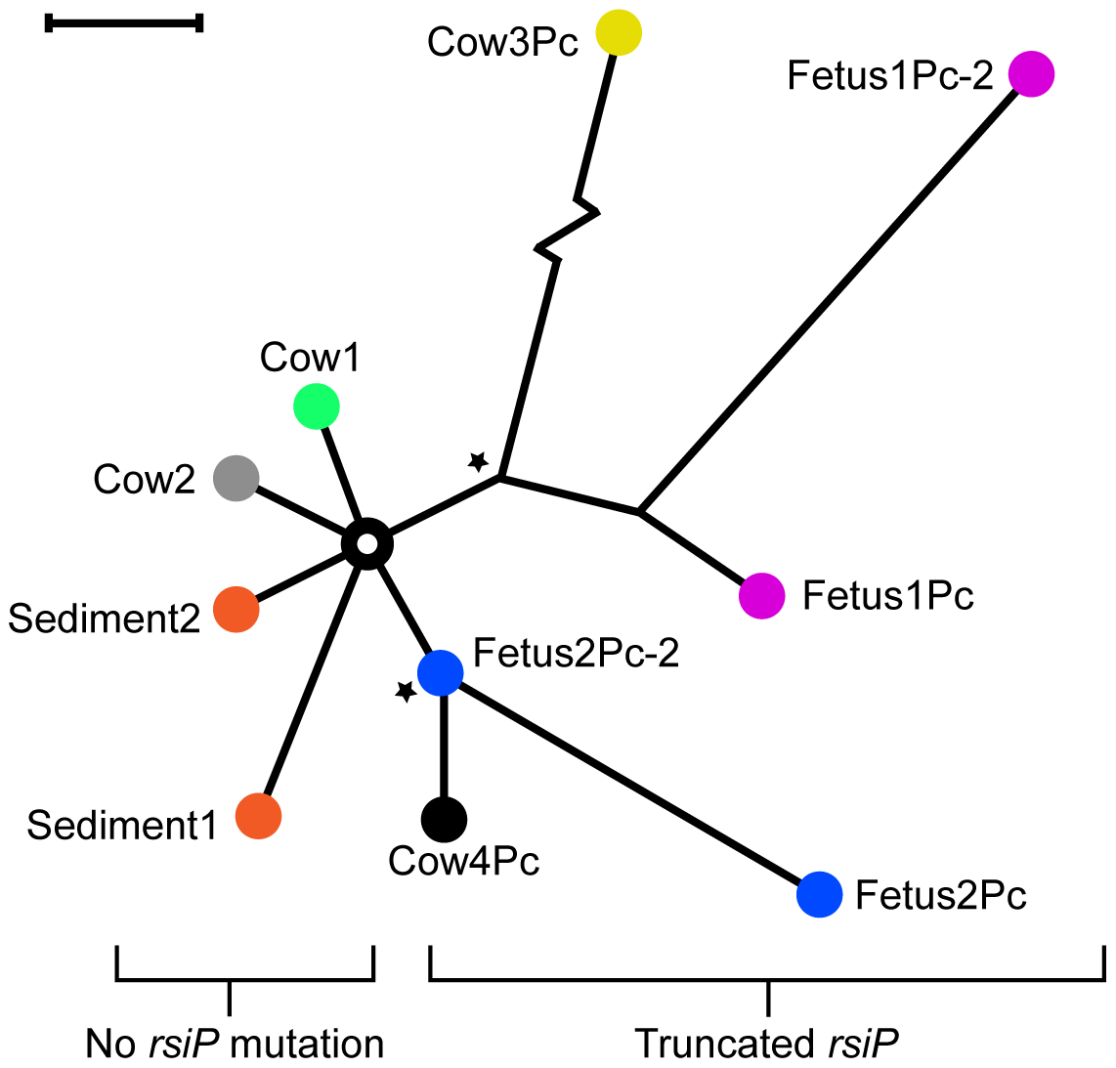
The relationship between the mutational profiles of different isolates in this study. The dendrogram was made by identifying and concatenating all SNPs and indels in the chromosomes. The different colors represent different animals. For some animals, two independent isolates were recovered. The “Sediment” isolates were recovered from the presumed source of infection. Stars represent two different mutations in the gene *rsiP* that led to penicillin resistance. The Cow3Pc isolate showed 54 chromosomal mutations compared to Cow1 (indicated as a zigzag line). The black bar equals 1 bp difference.

Six isolates were sequenced, of which two were from untreated cows that had died (Cow1, Cow2), two were from penicillin-treated cows that had died (Cow3Pc, Cow4Pc) and two from fetuses aborted from penicillin-treated cows (Fetus1Pc and Fetus2Pc). The cows that aborted the fetuses recovered from the disease after treatment. All SNVs (mutations, insertions and deletions) in the genome were determined and were concatenated into one sequence per isolate and used to create a dendrogram representing SNV differences (Figure 1). Assuming each animal had been infected from the same source (the anthrax grave from the 1940s) each isolate had acquired 1–4 SNVs except Cow3Pc. This isolate had acquired a hypermutator phenotype and had over 60 differences in the genome. All mutations found in these six isolates were unique except two mutations in a gene previously implicated in acquisition of penicillin resistance that were present in more than one isolate (described in detail below). Information about the SNVs can be found in Table S1. Thus, the sequence of the theoretical source of infection could be reconstructed representing the central node in the dendrogram (Figure 1).

In order to locate the source of infection, water sediment samples were taken every 20 meters along the dredged ditches. *B. anthracis* was detected at the waterside of a recently produced, 10-meter wide, indentation along one ditch. After refined sampling, a region of approximately 8 meters was identified from strongly positive PCR signals (data not shown). Cow footprints indicated that this area had clearly been a main drinking spot in this part of the pasture. Two soil samples that had shown positive PCR-results were cultivated on modified PLET agar which is a semi-selective medium for *B. anthracis* [24]. Two isolates were recovered (Sediment1 and Sediment2) and genome-sequenced using the MiSeq platform (Illumina). The mutation profiles were identified as for the previous animal isolates. The sediment isolates also carried 1–2 unique SNVs not found in the hypothetical ancestor (Figure 1) and they had no mutations in genes known to regulate antibiotic resistance.

### Genetic Heterogeneity within the Animals

The presence of different mutations in the isolates from the two closely located sediment samples suggested that there were heterogeneous SNVs in the source of infection. We had access to an additional, independent isolate from each of the two penicillin-treated, and later aborted, fetuses. Thus, two isolates were obtained from the first fetus. The first isolate (described above) came from the heart sack (Fetus1Pc) and the second from an abdominal aspiration (Fetus1Pc-2). Similarly, two isolates were obtained from the second fetus. The first isolate (described above) came from an abdominal aspiration (Fetus2Pc) and the second from the thorax (Fetus2Pc-2). The sequencing was performed on the MiSeq platform (Illumina) and analyzed as for the previous isolates. The paired isolates, Fetus1Pc and Fetus1Pc-2, both contained the resistance gene mutation and a second unrelated mutation as well as 1 respectively 4 unique mutations. The second pair of isolates, Fetus2Pc and Fetus2Pc-2 shared the resistance gene mutation but Fetus2Pc contained three additional SNVs (Figure 1). In conclusion, the mutation profile of independent isolates from the same animal were different and the number of mutations were in the same range as when comparing isolates from different animals or different isolates from the presumed source of infection.

This heterogeneity has profound consequences for the interpretation of the SNV dendrogram. These data imply that we are looking at both clonal mutations (present in the whole population) and subclonal ones (present in a subpopulation). The subclonal mutations are of limited phylogenetic value. We therefore wanted to quantify the penetration of the identified mutations in the sampled population. We performed amplicon sequencing on regions with the identified mutations using a crude DNA preparation made from the organ from which the isolate came. The result showed that the relative occurrence of the SNVs we had identified were in the range between fully clonal (∼100%) to below our limits of detection (the sequencing error rate makes it difficult to detect variants below 1%) (Table 1). Interestingly, the penetrance of mutations in the animals where two tissues had been sampled were different, suggesting there are spatial variations within the animal.

**Table 1:** Results of the mutation penetrance study.

Target (isolate)	Mutationnumber	DNA sample	Mutation positionin Cow1 genome	Total no of readscovering position	% of reads supportingthe mutation
Fetus2Pc	1	Fetus2Pc	432,614	46,895	40.20
Fetus2Pc	1	Fetus2Pc-2	432,614	113,661	0.18
Fetus2Pc	2	Fetus2Pc	2,370,360	30,500	21.64
Fetus2Pc	2	Fetus2Pc-2	2,370,360	76,229	0.42
Fetus2Pc	3	Fetus2Pc	1,594,715	29,359	25.50
Fetus2Pc	3	Fetus2Pc-2	1,594,715	41,409	3.33
Fetus1Pc-2	1	Fetus1Pc	3,642,409	77,515	0.04
Fetus1Pc-2	1	Fetus1Pc-2	3,642,409	7,831	0.08
Fetus1Pc-2	2	Fetus1Pc	2,022,492	114,584	0.27
Fetus1Pc-2	2	Fetus1Pc-2	2,022,492	53,538	0.26
Fetus1Pc-2	3	Fetus1Pc	2,363,596	47,247	0.22
Fetus1Pc-2	3	Fetus1Pc-2	2,363,596	34,425	0.21
Fetus1Pc andFetus1Pc-2	1	Fetus1Pc	3,705,779	60,996	98.79
Fetus1Pc andFetus1Pc-2	1	Fetus1Pc-2	3,705,779	56,783	99.92

The mutation areas were amplified with PCR and the amplicons were sequenced on the Illumina MiSeq. The background error levels were between 0.01–0.5% and were estimated by quantifying errors in positions surrounding the mutations.

### Mutation Rate Analysis

Mutations can arise both *in vivo* and during *in vitro* cultivation. The *in vitro* mutational rate of *B. anthracis* has been estimated to be 5.2×10^−10^ mutations/bp/generation [25]. This implies that limited expansions of colonies *in vitro* should have a low probability to produce new SNVs. To confirm this experimentally, we performed a sequential passage study. A single colony from a blood agar plate was transferred onto three new blood-agar plates and incubated at 37°C overnight. Three parallel passage studies were then performed. A single colony from each of the three plates was transferred onto new agar plates and this procedure was repeated for a total of ten passages. DNA was extracted from these three colonies at passages five and ten and from the starting colony. MiSeq (Illumina) sequencing was used to determine the mutation profiles. We could not detect any mutations in any of the three samples after five passages. However, after ten passages, two out of the three colonies showed one mutation each at different sites. Thus, the frequency of mutation during expansion of a colony (approximately 24 division cycles) was ∼0.1 per 24 generations (8.3×10^−10^ mutations/bp/generation), which is similar to the previously reported value [25]. This strongly indicates that in our experiment, the limited *in vitro* growth did not have a major impact on the *in vivo* mutation rate analysis.

A cow contains 50–85 ml of blood per kg body weight [26]. A cow weighing 600 kg would then contain 30–50 liters of blood. In the late stages of the disease, 10–100 million bacteria per ml blood can be present, corresponding to approximately 38–42 division cycles of exponential growth [27]. Thus, the *in vivo* mutation rate seems to be in the range of 1–2 mutations per 38–42 cell divisions (5–10×10^−9^ mutations/bp/generation). This is a factor 10–20 times higher than what is observed *in vitro* for either *B. anthracis* [25] or other species [8], [28]. One explanation could be that the *in vivo* mutation rate is higher than the *in vitro* rate. Alternatively, some of the mutations could have been inherited through heterogeneity in the infection dose. This heterogeneity may have been accumulated during growth in the animals preceding these ones in the infectious chain and the source may also have been a mixture of spores from several carcasses.

One of the resistant isolates, Cow3Pc, showed a hypermutator phenotype. Among the identified mutations, there was a truncation of *mutL*, a component of the mismatch reparation system. Ultimately, this isolate is most likely to be associated with loss of fitness. Hypermutator phenotypes during outbreaks have been observed before [29]. The *in vivo* mutation rate was elevated approximately 30–60 times. This error rate implies that mutations arise every division cycle and indeed, we found high-quality, ambiguous nucleotide positions in the genome assembly. These indicate that heterogeneity also arose during the cultivation of this isolate prior to DNA sequencing.

### Microevolution of Antibiotic Resistance within the Treated Animals

As a routine procedure, the minimum inhibitory concentrations (MIC) of different antibiotics were determined for the isolates recovered from the outbreak. Beta-lactamase production was also tested (Table 2). Isolates from the untreated cows were susceptible to the beta-lactams penicillin and ampicillin and also to tetracycline, ciprofloxacin and chloramphenicol (Cow1 and Cow2). Susceptibility to the other antimicrobials tested cannot be determined because interpretive criteria are lacking. However, with the exception of trimethoprim, to which *B. anthracis* is considered intrinsically resistant [15], MICs for gentamicin, kanamycin, streptomycin, erythromycin and clindamycin were low and do not indicate acquired resistance. Beta-lactamase production was not detected in these isolates. In contrast, isolates from penicillin-treated cows and isolates from aborted fetuses of treated cows had a penicillin-resistant phenotype with high MICs for penicillin and ampicillin and confirmed beta-lactamase production (Cow3Pc, Cow4Pc, Fetus1Pc and Fetus2Pc). MICs for the other antimicrobials tested did not deviate from MICs of the penicillin susceptible isolates.

**Table 2:** MIC (mg/L) and beta-lactamase production in isolates from untreated cows (Cow1 and Cow2), penicillin-treated cows (Cow3Pc and Cow4Pc), and aborted fetuses of penicillin-treated cows (Fetus1Pc and Fetus 2Pc).

MIC (mg/L)												
Isolate	Pc(≤0.12)	Am(Na)	Cip(≤0.25)	Tc(≤1)	Gm(Na)	Km(Na)	Sm(Na)	Em(Na)	Cl(Na)	Tm(Na)	Cm(≤8)	Beta-lactamase
Cow1	≤0.03	≤0.12	0.12	0.25	0.5	0.5	2	≤0.25	1	>32	1	no
Cow2	≤0.03	≤0.12	0.06	≤0.12	0.5	≤0.25	4	≤0.25	≤0.25	16	1	no
Cow3Pc	>4	>16	0.06	≤0.12	0.5	4	4	0.5	0.5	>32	4	yes
Cow4Pc	>4	>16	0.06	≤0.12	0.5	2	4	0.5	0.5	>32	4	yes
Fetus1Pc	4	8	0.06	≤0.12	0.25	2	≤1	0.5	0.5	>32	2	yes
Fetus2Pc	>4	>16	0.06	≤0.12	0.5	0.5	4	≤0.25	≤0.25	>32	2	yes

Na: not available; Pc: penicillin; Am: ampicillin; Cip: ciprofloxacin; Tc: tetracycline; Gm: gentamicin; Km: kanamycin; Sm: streptomycin; Em: erythromycin; Cl: clindamycin; Tm: trimethoprim; Cm: chloramphenicol.

Breakpoints for susceptibility according to CLSI (2010) [33] are given inside brackets when available.

Penicillin resistance can be induced *in vitro* [30] and all thus far sequenced *B. anthracis* have two antibiotic resistance genes, *bla1* and *bla2*, that encode a penicillinase and cephalosporinase respectively [31]. However, the genes are normally inactive [31]. In closely related species, the *bla* genes are controlled by an extracytoplasmic sigma factor (ECF) called *sigP* that, in turn, is repressed by an anti-sigma factor *rsiP*. The sigma factor and its anti-sigma factor are present in *B. anthracis* as well, but normally do not respond to drug exposure [31]. Studies of resistant *B. anthracis* isolates are sparse although there is a report of a mutation in the anti-sigma factor *rsiP* gene giving rise to a frameshift mutation. This resulted in a constitutive transcription from the *bla* genes [31].

The genomic data for the resistant isolates in our study were examined and in two isolates, Fetus1Pc and Cow3Pc, we found the exact same frameshift mutation in the *rsiP* gene (corresponding to amino acid 6 of 275) as reported by Ross et al. [31]. This region is a homopolymer one and may therefore be a hotspot for insertion/deletion events. For the Cow4Pc and Fetus2Pc isolates, the mutation was located further downstream in the *rsiP* gene with an inserted G, also causing a frameshift mutation (at a position corresponding to amino acid 163 of 275). The Fetus1Pc-2 and Fetus2Pc-2 isolates shared the same *rsiP* gene mutations as their paired isolate from the same animal.

### Counteracting Mutations in Penicillin Resistant Strains

A study by Ross et al. suggests that constitutive expression of wild type *sigP* can be detrimental to *B. anthracis* growth [31]. They found an amino acid change in *sigP* that presumably led to reduced activity. In our resistant strains, the *sigP* activity seemed to be tolerated. However, in the case of the isolates from one of the fetuses, we made some peculiar observations. In one out of two independent DNA preparations from the Fetus1Pc isolate, we found a nonsense mutation in the *sigP* gene at amino acid position 105 out of 179. It turned out that the isolate was impure and approximately 10% of the frozen stock contained this inactivation of *sigP* (re-isolated as Fetus1Pc-counteracting). This subpopulation had lost their resistant phenotype (data not shown). Interestingly, another, unrelated *sigP* mutation (amino acid position 91 out of 179) was also found in the Fetus1Pc-2 isolate (the second isolate coming from the same animal). Amplicon sequencing showed that the penetrance of both mutations were below detection levels in the tissue sample. The fact that we readily isolated subpopulations with these counteracting mutations, despite apparent low frequencies in the tissue samples, suggests that these subpopulations had an advantage during the isolation process.

### Transcriptome Analysis of the Resistant and Susceptible Isolates

**Figure 2:**
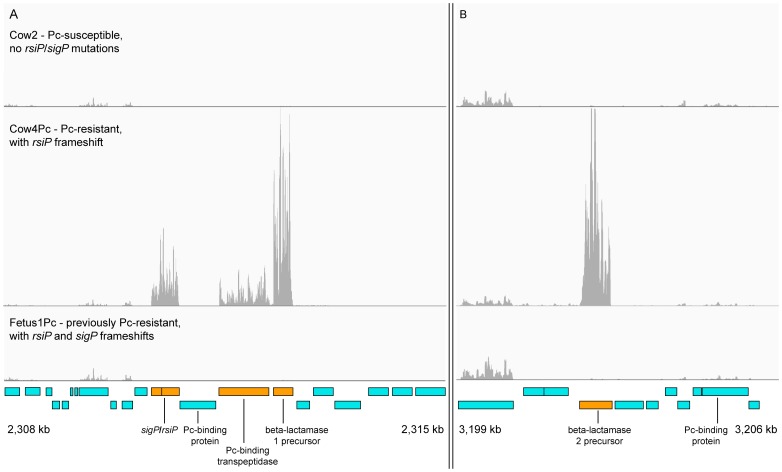
Transcriptome analysis of genes involved in penicillin resistance. The three horizontal panels show reads for three samples mapped to the genome. The upper and middle panels show that the mutation in *rsiP* led to a profound up-regulation of five genes: A) *rsiP* anti-sigma factor (BAPAT_2394), *sigP* sigma factor (BAPAT_2393), beta-lactamase 1 (BAPAT_2397), penicillin-binding protein transpeptidase (BAPAT_2396) and shown in B) the beta-lactamase 2 (BAPAT_3350). The lower panel shows that the counteracting *sigP* mutation found in a subclonal population in one animal had quenched expression completely.

Given that the resistance mutations were located in an anti-sigma factor, we wanted to measure the effect at a genome-wide transcriptional level. Thus, we performed an RNAseq analysis on a susceptible isolate (Cow2), a resistant one (Cow4Pc), and one that had lost the resistance phenotype by a compensatory mutation (Fetus1Pc-counteracting). The results showed that the major transcriptional effect was limited to five genes: *rsiP* anti-sigma factor (BAPAT_2394), *sigP* sigma factor (BAPAT_2393), beta-lactamase 1(BAPAT_2397), beta-lactamase 2 (BAPAT_3350) and penicillin-binding protein transpeptidase (BAPAT_2396). These genes were significantly upregulated 400–800 fold as determined with the Cuffdiff 2 software [32]. The full table is available as Table S2. The transcriptome mapping for these genes is shown in Figure 2 which shows the massive regulatory effect that the *rsiP* mutation has on beta-lactamase expression. It also shows that the counteracting mutation in *sigP* completely abolishes the acquired expression.

## Discussion

Our results demonstrate the power of genome analysis during a very confined outbreak situation as well as the limitations involved in resolving individual transmissions. In the case studied here, it is likely that all isolates passed only once through an animal. Therefore we hoped that we could use mutational profiles to link and compare the outbreak isolates to the environmental isolates from the source of infection. However, our studies showed that each cow had an intra-host heterogeneity at time of death and that different isolates from the same dead animal had a similar number of mutational differences as found when comparing isolates between different dead animals. Further, environmental isolates from the presumed source of infection were also heterogeneous and this heterogeneity was likely transmitted, as outlined in detail below.

**Figure 3:**
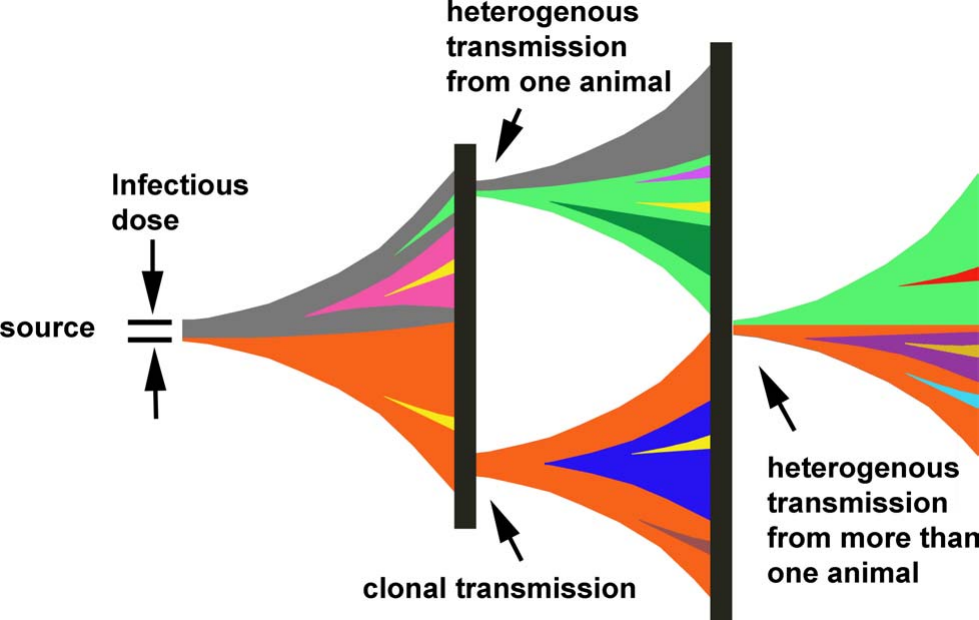
A model for how heterogeneity is transmitted through the infectious dose. Colors represent different mutation profiles in a subclone (i.e., a subclonal mutation present in a fraction of the population). Heterogeneity in the preceding animal in the infectious chain may lead to different mutations being transmitted in different animals even though they were infected from the same carcass. The vertical black boxes represent the soil/sediment and can be seen as a randomizer mixing the spores which will infect next host. A clonal mutation (i.e., one present in the whole population) is valuable for tracing purposes but may be subclonal in the preceding case. Thus, it is evident that there is a need to analyze several isolates to determine the clonality of the observed mutations and to fully comprehend the transmission chain.

This study illustrates several pitfalls that must be taken into account when doing high-resolution tracing with NGS. Most importantly, an isolate is only a randomly chosen representative from the population. Several isolates must be studied to judge whether the mutations are truly representative for the whole population. This has consequences for the interpretations of susceptibility testing, since small subpopulations with resistance mutations may exist during an infection. Regional differences in different parts of the host body may also lead to failure to detect a resistant subpopulation. Not all of the observed heterogeneity is necessarily a result of mutations during bacterial growth in that animal. Heterogeneity in the actual dose that the disease was acquired from likely contributes to the observed heterogeneity. In addition, variations in the size of the infectious dose can also, through founder effects, affect the amount of heterogeneity transferred to the studied case. Transmission of heterogeneity through the infectious dose may also contribute to cumulative accumulation of higher levels of heterogeneity. A model of how transmissions could occur is depicted in Figure 3. Another consequence of this inherited heterogeneity is that it may place a limitation on the resolution achievable in outbreak analysis with whole genome sequencing.

Our data imply a scenario in which penicillin resistance was present in a small subclonal population. Under selection pressure during antibiotic treatment, it expanded to become the major clone. This second expansion in the same animal explains why the resistant isolates seemed to contain more mutations than the susceptible strains (Figure 1). If the heterogeneity and bacterial load become high enough, resistance may and probably will arise. In this studied case, the infectious dose may have been both high (given that so many animals were simultaneously infected), and heterogeneous (as suggested by the observed differences in environmental isolates). These two factors may have facilitated the development of *in vivo* penicillin resistance. In humans, milder cases will likely not reach high enough bacterial load for resistance to develop. However, our results indicated that the risk for emergence of resistance in cases with systemic involvement may be notable, especially if the source of infection was heterogeneous.

## Materials and Methods

No experimental animals have been used. Samples were only collected from dead animals in a natural disease outbreak and no animal was killed for the purposes of this study. The samples were taken by a veterinarian with the primary purpose to confirm diagnosis so that remaining animals could be treated appropriately.

### Procedures

The isolates were tested for susceptibility to antibiotics by determination of MIC with broth microdilution using VetMIC test-kits (SVA, Uppsala Sweden) according to CLSI [33]. *Staphylococcus aureus* ATCC 29213 was used as the quality-control strain. In addition, all isolates were tested for beta-lactamase production using the nitrocefin test as described by Hernàndez-Guinàt [15].

The genome of the reference strain (the first isolated isolate, named Cow1), and the genomes of two isolates that were penicillin resistant, were sequenced using the Roche 454 GS FLX+ (Roche, Basel, Switzerland) and the Illumina HiSeq 2000 platform (Illumina, San Diego, CA, USA). The sequence reads were assembled in GS Reference Mapper (Roche) using the *B. anthracis* Ames Ancestor (Genbank Accession NC_007530) as reference. The gaps between contigs were closed using Sanger sequencing to produce one chromosome and two plasmids. The seven following isolates (Sediment1, Sediment2, Cow2, Cow4Pc, Fetus2Pc, Fetus1Pc-2, and Fetus2Pc-2) were prepared using the Nextera XT kit (Illumina) and sequenced on the MiSeq platform (Illumina) using 2×250 bp sequencing settings. The reads were mapped with Consed/cross-match to the finished Cow1-genome and Consed was set up to show all highly discrepant positions [34]. After manual sorting of the positions, this yielded a list of all significant differences between the Cow1 reference and the other sequenced isolates. The finished genome of isolate Cow1 along with MiSeq-reads for Cow1 and the other nine isolates have been deposited to Genbank under BioProject accession number PRJNA217316. The accession number for the Cow1 chromosome and plasmids are CP006742, CP006743 and CP006744, respectively.

For the transcriptome sequencing, total RNA was extracted using the Ambion® RiboPure™-Bacteria Kit (Life technologies, Carlsbad, CA, USA), rRNA-depleted using the Ribo-Zero™ Magnetic Kit (Gram-Positive Bacteria) (Epicentre Biotechnologies, Madison, WI, USA) and then prepared for MiSeq-sequencing with the ScriptSeq™ v2 RNA-Seq Library Preparation Kit (Epicentre). FASTQ-files from the 1×50 bp-run have been deposited to Genbank under BioProject accession number PRJNA217316. The reads were aligned to the annotated coding regions of the Cow1 isolate by using Bowtie 2 with the ‘very-sensitive’ setting [35]. Significant transcription-level differences were determined using Cufflinks/Cuffdiff [32].

To investigate the intra-animal heterogeneity using amplicon NGS, primers were designed to amplify the regions containing the determined SNPs/indels. PCR was performed using these primers and samples used were the original DNA-extractions from the samples analyzed during the outbreak. Samples were used together with the primers for the mutation that the DNA-sequencing had shown to exist in that sample. Different samples from the same animal were also analyzed for all mutations found in isolates from that animal. The reads were aligned to FASTA-files of the ∼2 kb-regions of the mutations using the Bowtie2 aligner. The ratio of a certain mutation was calculated by counting the number of reads supporting the two variants.

For the *in vitro* mutation-rate experiment, an isolate was cultivated on a horse-blood agar plate overnight at 37°C after which a single colony was spread onto three new plates. After another overnight incubation at 37°C, a single colony from each plate was spread onto a new plate. This process was repeated 9 times for at total of 10 passages. From the starting plate and from each of the three plates after passages 5 and 10, a single colony was used to inoculate 5 ml of medium. The cultures were then extracted for DNA and the DNA sequenced on the MiSeq (Illumina). The mutation rate was estimated by assuming a probability of 0.333 (one out of three) that the bacteria had not acquired a mutation after 10 passages. Thus, if the probability of acquiring a mutation in each passage is x, the probability of not acquiring a mutation is (1–x) and for all 10 passages (1–x)^10^. Thus, x = 1–0.333^1/10^. A colony was estimated to contain approximately 16 million bacteria, corresponding to 24 division cycles of exponential growth [36], [37].

More detailed procedures regarding, e.g., DNA-extractions, cultivations and DNA-sequencing, can be found in Text S1.

## Supporting Information

Table S1 The identified SNVs (SNPs and indels) across the 10 isolates and their coordinates in the Cow1 reference genome.
(PDF)

Table S2 RNAseq data results from Cuffdiff 2 software complemented with annotations.
(PDF)

Text S1 Detailed procedures.
(PDF)
