# Supplementary material for: Microevolution during an Anthrax Outbreak Leading to Clonal Heterogeneity and Penicillin Resistance
Source: PLoS One. 2014 Feb 13;9(2):e89112. doi: 10.1371/journal.pone.0089112 (PMC3923885; doi:10.1371/journal.pone.0089112)
Supplement: Table S1 — The identified SNVs (SNPs and indels) across the 10 isolates and their coordinates in the Cow1 reference genome. (PDF) [file pone.0089112.s001.pdf]

Supplementary table S1. The identified SNVs (SNPs and indels) across the 10 isolates and their coordinates in the Cow1 reference genome.

Annotations covering the SNV and the effect the SNV has on the gene product are also shown
(letters indicate amino acids, AA = amino acids)
For clarity, SNVs shared by two or more isolates are highlighted and the more than 60 mutations found only in the hypermutating Cow3Pc have been omitted.

SNV type	Coord in Cow1	Cow1	Cow2	Cow3Pc	Cow4Pc	Fetus1Pc	Fetus2Pc	Sediment 1	Sediment 2	Fetus1Pc-2	Fetus2Pc-2
SNP	432,614	C	C	C	C	C	T	C	C	C	C
SNP	671,082	T	T	T	T	T	T	T	C	T	T
deletion	1,594,715	A	A	A	A	A	-	A	A	A	A
SNP	1,773,112	A	A	A	A	A	A	T	A	A	A
SNP	2,022,492	C	C	C	C	C	C	C	C	T	C
deletion	2,308,984	A	A	A	A	A	A	A	A	-	A
SNP	2,309,056	C	C	C	C	T	C	C	C	C	C
deletion	2,309,285	A	A	-	A	-	A	A	A	-	A
insertion	2,309,740	-	-	-	G	-	G	-	-	-	G
SNP	2,363,596	G	G	G	G	G	G	G	G	T	G
SNP	2,370,360	A	A	A	A	A	G	A	A	A	A
SNP	2,413,725	A	G	A	A	A	A	A	A	A	A
insertion	2,758,175	-	C	C	C	C	C	C	C	C	C
deletion	3,353,320	T	T	T	-	T	T	T	T	T	T
SNP	3,642,409	A	A	A	A	A	A	A	A	C	A
SNP	3,705,779	A	A	A	A	G	A	A	A	G	A
SNP	4,003,275	G	G	G	G	G	G	T	G	G	G

**Coord in Cow1**	**Annotation**	**Effect on protein**
432,614	DNA-3-methyladenine glycosylase II	A --> V
671,082	Ribokinase	silent mutation
1,594,715	-	
1,773,112	Multicopper oxidase family protein	N --> Y
2,022,492	Isoleucyl-tRNA synthetase 2	P --> L
2,308,984	RNA-polymerase sigma-70 factor (sigP)	Frameshift, creates 91 AA product (product usually 179 AA)
2,309,056	RNA-polymerase sigma-70 factor (sigP)	Stop codon, creates 105 AA product (product usually 179 AA)
2,309,285	RNA-polymerase sigma-70 factor (rsiP)	Frameshift, creates 12 AA product (product usually 275 AA)
2,309,740	RNA-polymerase sigma-70 factor (rsiP)	Frameshift, creates 163 AA product (product usually 275 AA)
2,363,596	Acetoacetyl-CoA synthase	W -- > C
2,370,360	DNA-binding response regulator	silent mutation
2,413,725	Group-specific protein	silent mutation
2,758,175	-	
3,353,320	Ribonuclease-triphosphate reductase	Frameshift, creates 100 AA product (product usually 152 AA)
3,642,409	Signal recognition particle protein	D --> G
3,705,779	Peptidase U4 sporulation factor SpoIIGA	I --> T
4,003,275	bifunctional protein folD	T --> K
