# Supplementary material for: Microevolution during an Anthrax Outbreak Leading to Clonal Heterogeneity and Penicillin Resistance
Source: PLoS One. 2014 Feb 13;9(2):e89112. doi: 10.1371/journal.pone.0089112 (PMC3923885; doi:10.1371/journal.pone.0089112)
Supplement: Table S2 — RNAseq data results from Cuffdiff 2 software complemented with annotations. (PDF) [file pone.0089112.s002.pdf]

Supplementary Table S2. RNAseq data results from Cuffdiff 2 software complemented with annotations.

Only comparisons reported significant by the software are shown and sorted in decreasing fold change.
The data are presented in three subtables as Cuffdiff 2 analyzes samples pair wise.
FPKM is fragments per kilobase of gene per million fragments mapped

Cow2 versus Cow4Pc

locus	FPKM Cow2	FPKM Cow4Pc	log2 (fold_change)	p_value	significant	Annotation
BaPAT_2396:0-2136	4,4	3351,4	9,6	0	yes	Penicillin-binding protein transpeptidase
BaPAT_2397:0-893	39,0	26561,2	9,4	0	yes	Beta-lactamase 1 precursor
BaPAT_3350:0-793	10,2	6300,8	9,3	0	yes	Beta-lactamase 2 precursor
BaPAT_2394:0-826	13,7	8271,3	9,2	0	yes	RNA-polymerase sigma-70 factor (rsiP)
BaPAT_2393:0-548	16,4	8648,2	9,0	0	yes	RNA-polymerase sigma-70 factor (sigP)
BaPAT_4070:1-685	19,9	140,5	2,8	1.0534e-07	yes	Membrane protein
BaPAT_4071:377-763	25,2	167,4	2,7	0,000102906	yes	Membrane protein
BaPAT_2399:11-985	27,1	98,7	1,9	0,000111403	yes	Transcriptional regulator
BaPAT_1713:8-1275	208,6	634,3	1,6	4.24967e-05	yes	Proton/sodium-glutamate symporter
BaPAT_3506:15-1858	262,9	92,8	-1,5	0,000124827	yes	Anaerobic ribonucleoside-triphosphate
BaPAT_1857:0-1017	618,1	198,0	-1,6	2.78724e-05	yes	Cytochrome d ubiquinol oxidase, subunit II
BaPAT_5398:1-1056	65,3	17,3	-1,9	0,00014685	yes	Iron compound ABC transporter, permease protein
BaPAT_1856:1-1407	321,8	83,6	-1,9	1.47685e-06	yes	cytochrome d ubiquinol oxidase subunit I
BaPAT_2263:14-1618	33,7	8,3	-2,0	0,000113674	yes	Oxalate:formate antiporter
BaPAT_5062:9-921	279,0	57,5	-2,3	1.66435e-07	yes	SAM-dependent methyltransferase
BaPAT_0792:11-909	147,9	29,6	-2,3	9.53748e-07	yes	N-acetylmuramic acid 6-phosphate etherase

Cow2 versus Fetus1Pc

locus	FPKM Cow2	FPKM Fetus1Pc	log2 (fold_change)	p_value	significant	Annotation
BaPAT_1840:2-996	11,1	242,1	4,4	1.73541e-11	yes	S-layer protein
PAT_pXO1_0103:12-2100	2,7	40,6	3,9	1.11244e-06	yes	hypothetical protein
BaPAT_2053:8-708	33,9	422,5	3,6	5.08636e-09	yes	ScdA protein
PAT_pXO1_0045:2-686	11,4	98,7	3,1	0,000100281	yes	hypothetical protein
BaPAT_1616:1-679	124,3	1065,5	3,1	9.01002e-09	yes	hypothetical protein
PAT_pXO1_0080:9-1431	5,1	43,5	3,1	5.76757e-05	yes	Type II secretion system protein
PAT_pXO1_0081:5-933	8,6	59,9	2,8	0,000304735	yes	membrane protein
BaPAT_0661:5-893	30,5	207,5	2,8	1.24821e-05	yes	Methyl-accepting chemotaxis protein
BaPAT_0535:2-1954	16,8	105,5	2,7	1.19576e-05	yes	Methyl-accepting chemotaxis protein
BaPAT_4056:22-1236	14,9	83,3	2,5	0,000197801	yes	Maltose-maltodextrin ABC-related solute binding prot
BaPAT_1622:0-798	1610,6	8978,8	2,5	7.36242e-11	yes	Flagellin
BaPAT_3152:8-1984	26,3	138,3	2,4	2.8337e-05	yes	Methyl-accepting chemotaxis protein
BaPAT_1922:3-1946	26,1	131,4	2,3	5.29761e-05	yes	Methyl-accepting chemotaxis protein
PAT_pXO1_0104:6-3589	9,2	45,5	2,3	0,000166968	yes	Reticulocyte binding protein
BaPAT_3534:0-217	992,6	4604,0	2,2	0,000206257	yes	hypothetical protein
BaPAT_5063:0-1658	89,2	399,4	2,2	9.50964e-06	yes	Methyl-accepting chemotaxis protein
BaPAT_1608:6-917	105,6	448,6	2,1	0,000109175	yes	Chemotaxis protein
BaPAT_1578:1-1308	63,4	257,5	2,0	0,000217307	yes	hypothetical protein
BaPAT_2844:16-1196	111,8	401,8	1,8	0,000294883	yes	SH3 domain-containing protein
BaPAT_2066:1-730	216,8	37,4	-2,5	8.63573e-05	yes	hypothetical protein
BaPAT_2257:0-240	1475,5	232,5	-2,7	8.56233e-05	yes	ArsR family transcriptional regulator
BaPAT_5398:1-1056	67,6	10,5	-2,7	0,000157203	yes	Iron compound ABC transporter, permease prot
BaPAT_1314:1-770	96,5	14,4	-2,7	0,000151124	yes	Group-specific protein
BaPAT_1025:3-2152	26,0	3,8	-2,8	0,000180298	yes	membrane protein
BaPAT_1494:0-379	682,0	92,6	-2,9	8.88671e-06	yes	Spore coat protein D
BaPAT_1163:0-468	293,2	37,8	-3,0	1.83421e-05	yes	Spore coat protein Y
BaPAT_3436:6-329	417,6	36,5	-3,5	4.03953e-05	yes	hypothetical protein
BaPAT_5062:13-921	290,2	21,1	-3,8	1.60195e-09	yes	SAM-dependent methyltransferase
BaPAT_0471:8-649	123,0	6,8	-4,2	5.23748e-05	yes	Integral membrane protein

Cow4Pc versus Fetus1Pc

locus	FPKM Cow4Pc	FPKM Fetus1Pc	log2 (fold_change)	p_value	significant	Annotation
BaPAT_2053:8-708	26,6	408,5	3,9	8.6076e-10	yes	ScdA protein
BaPAT_1840:7-996	19,2	235,5	3,6	1.6285e-08	yes	S-layer protein
PAT_pXO1_0045:2-686	9,4	95,5	3,3	4.45e-05	yes	hypothetical protein
BaPAT_0792:12-916	28,5	230,8	3,0	2.11273e-06	yes	N-acetylmuramic acid 6-phosphate etherase
PAT_pXO1_0081:5-933	7,3	57,9	3,0	0,000132249	yes	Membrane protein
BaPAT_1616:1-679	136,6	1030,1	2,9	9.06003e-07	yes	hypothetical protein
BaPAT_3469:1-1308	8,2	61,1	2,9	3.05003e-05	yes	Molybdopterin molybdochelatase
BaPAT_4385:10-624	11,9	87,3	2,9	0,000345023	yes	Nitroreductase
BaPAT_3470:1-1017	7,9	52,1	2,7	0,000259462	yes	Molybdopterin biosynthesis protein MoeB
BaPAT_3471:8-799	10,9	70,8	2,7	0,000296699	yes	Formate/nitrite transporter
BaPAT_3506:9-1858	89,8	572,8	2,7	2.94354e-06	yes	Anaerobic ribonucleoside-triphosphate reductase
BaPAT_0661:5-893	32,5	200,6	2,6	3.819e-05	yes	Methyl-accepting chemotaxis protein
PAT_pXO1_0080:9-1431	7,1	42,1	2,6	0,00027184	yes	Type II secretion system protein
BaPAT_4845:1-1016	314,1	1764,7	2,5	1.14798e-05	yes	Cytochrome d ubiquinol oxidase subunit II
BaPAT_0535:2-1959	18,5	101,7	2,5	7.23441e-05	yes	Methyl-accepting chemotaxis protein
BaPAT_1922:3-1946	24,1	127,0	2,4	8.12034e-05	yes	Methyl-accepting chemotaxis protein
BaPAT_5039:6-2002	12,8	64,4	2,3	0,000288948	yes	Methyl-accepting chemotaxis protein
BaPAT_5063:0-1658	93,9	386,2	2,0	0,000335786	yes	Methyl-accepting chemotaxis protein
BaPAT_1533:3-14935	38,2	7,4	-2,4	4.25031e-05	yes	Repeat domain protein
BaPAT_2257:0-260	1115,0	175,8	-2,7	8.03663e-05	yes	ArsR transcriptional regulator
BaPAT_4070:1-685	136,5	20,9	-2,7	8.26353e-05	yes	Membrane protein
BaPAT_1163:0-440	346,9	40,9	-3,1	6.56903e-06	yes	Spore coat protein Y
BaPAT_3675:485-1156	105,6	11,5	-3,2	3.71097e-05	yes	hypothetical protein
BaPAT_4534:0-193	5194,7	477,2	-3,4	1.5932e-07	yes	Spore germination protein gerE
BaPAT_3436:7-342	367,6	32,3	-3,5	2.4515e-05	yes	hypothetical protein
BaPAT_1494:0-399	1075,1	82,3	-3,7	5.48479e-09	yes	Spore coat protein D
BaPAT_2394:0-826	8035,7	20,7	-8,6	0	yes	RNA-polymerase sigma-70 factor (rsiP)
BaPAT_2393:0-548	8401,9	17,7	-8,9	0	yes	RNA-polymerase sigma-70 factor (sigP)
BaPAT_2396:0-2136	3255,9	5,4	-9,2	0	yes	Penicillin-binding protein transpeptidase
BaPAT_3350:0-793	6121,3	9,7	-9,3	0	yes	Beta-lactamase 2 precursor
BaPAT_2397:0-893	25804,6	37,4	-9,4	0	yes	Beta-lactamase 1 precursor
