# Supplementary material for: Microevolution during an Anthrax Outbreak Leading to Clonal Heterogeneity and Penicillin Resistance
Source: PLoS One. 2014 Feb 13;9(2):e89112. doi: 10.1371/journal.pone.0089112 (PMC3923885; doi:10.1371/journal.pone.0089112)
Supplement: Text S1 — Detailed procedures. (PDF) [file pone.0089112.s003.pdf]

Supplementary Text S1- detailed procedures

DNA-sequencing and mutation discovery

Ten ml of Luria-Bertani medium was inoculated with a colony of *B. anthracis* pre-cultivated on a horse-blood agar plate. After incubation overnight at 37°C, 1.5 ml of culture was centrifuged at 7,000 x *g* and DNA was prepared from the pellet using a MasterPure Gram positive kit (Epicentre Biotechnologies, Madison, WI, USA). To ensure that no *B. anthracis*-spores were in the sample, the DNA was filtered through an Ultrafree-MC 0.22 µm sterile filter (Merck Millipore, Billerica, MA, USA) before it was taken from the BSL-3 facility. The quality and quantity of the DNA was assessed on an agarose gel, a NanoDrop spectrophotometer (Thermo Scientific, NanoDrop Products, Wilmington, DE, USA) and a Qubit® dsDNA BR Assay Kit (Life technologies, Carlsbad, CA, USA). Sequencing on both the Roche 454 GS FLX+ and the Illumina HiSeq 2000 was performed by Science for Life Laboratory (Stockholm, Sweden). The 454-run used chemistry that enabled long read-lengths and it produced an average genome coverage of the Cow1, Cow3Pc, and Fetus1Pc isolates of 21x, 30x and 22.5x, respectively. The sequence reads were assembled in GS Reference Mapper (Roche, Basel, Switzerland) using the *B. anthracis* Ames Ancestor (Genbank Accession NC_007530) as reference. The gaps between contigs were closed using Sanger sequencing to produce one chromosome and two plasmids. To achieve a higher quality of the assembly, Illumina-reads were mapped to the assemblies to correct errors as these reads were of very high quality. The average coverage was 112x. The same procedure was used for Cow3Pc and Fetus1Pc to create three complete genomes. The finishing software was Consed v. 23.0 (1). Annotation was done with Glimmer 3.02 to find all open reading frames (2). A comparison of these with other annotated strains available from the NCBI, allowed an updated annotation for each anthrax genome used. The genomes were analyzed in MUMmer v.3.23 (3) using the '–show-snps' and 'show-diffs' arguments to find SNPs and differences between the genomes. The chromosomes differed in size from those in Cow1 with only one and four bases, respectively; these differences were due to single base insertions and deletions. This supports our assumption that the isolates originated from the same strain.

The seven isolates (Sediment1, Sediment2, Cow2, Cow4Pc, Fetus2Pc, Fetus1Pc-2, and Fetus2Pc-2) were sequenced using the same method of DNA-preparation as above but they were sequenced on the MiSeq platform (Illumina). One nanogram of DNA was used as starting material and prepared using the Nextera XT DNA Sample Preparation Kit (Illumina, San Diego, CA, USA). The sequencer performed paired-end 2 x 250 bp reads yielding an average coverage of 140x, 141x, 66x, 129x, 181x, 32x, and 155x for the seven isolates, respectively. The reads were then mapped with Consed/cross-match to the finished Cow1-genome and Consed was set up to show all highly discrepant positions. After manual sorting of the positions, this yielded a list of all significant differences between the Cow1 reference and the newly sequenced isolates. The finished genome of isolate Cow1 along with MiSeq-reads for Cow1 and the other nine isolates have been deposited to Genbank under BioProject accession number PRJNA217316. The accession number for the Cow1 chromosome and plasmids are CP006742, CP006743 and CP006744, respectively.

DNA extraction of soil samples and PCR analysis

A germination/enrichment step was added before the DNA isolation where a small amount of soil sample was incubated in 400 µl Brain-Heart-Infusion medium in a 1.5 mL reaction tubes for 4 hours at 37°C with shaking in a thermomixer at 500 rpm. To monitor the success of the extraction, an internal control of 40 spores of a *Bacillus thuringiensis* serovar kurstaki strain was added prior to incubation. The complete content of the tube was, after incubation, added to the PowerSoil® DNA Isolation Kit (MO BIO Laboratories, Inc., Carlsbad, CA, USA) and the DNA was extracted using the manufacturer's instructions.

A multiplex PCR was used for detection where a *B. thuringiensis*-specific target was used together with an anthrax-specific chromosomal marker from Wielinga et al.(4). This PCR system indicated both presence of anthrax-DNA and the success of DNA-extraction. Samples positive for DNA of *B. anthracis* were rerun in a three-plex PCR with the plasmid targets added (4). The samples were also tested for inhibition in a separate PCR tube where a PCR for a completely unrelated target was run along with a determined amount of DNA for that target. Although this was an external inhibition control, taken together with the internal extraction control in the other PCR tube it clearly showed that no PCR inhibition was present.

Transcriptome sequencing

Ten ml of Luria-Bertani-medium was inoculated with a colony of *B. anthracis* pre-cultivated on a horse-blood agar plate. During 37°C incubation, the OD_{600} values were monitored and 1 ml samples were taken when the cultures reached logarithmic growth phase (i.e., OD_{600} 0.4, ~2.5 h). The samples were centrifuged and pellets were resuspended in RNAlater® (Life Technologies) and stored at 4°C until RNA extraction. Extraction was made using the Ambion® RiboPure™-Bacteria Kit (Life technologies); this kit contains zirconia beads to facilitate the disruption of the cell walls. Genomic DNA was removed with the Ambion DNA-free™ reagents included in the kit. The extracted RNA was centrifuged through an Ultrafree-MC 0.22 µm sterile filter (Merck Millipore) to ensure sterility before moving the sample from the BSL-3 laboratory to a BSL-2 laboratory. Yields and RNA-qualities were assessed using the Agilent 2100 Bioanalyzer with the RNA 6000 Pico Kit (Agilent Technologies, Waldbronn, Germany). For all samples, yields were between 5 and 15 µg total RNA as measured with the Qubit. At this stage in the workflow, the ribosomal RNA peaks dominated the output from the Bioanalyzer as they make up the majority of total RNA in bacteria. Since we were not interested in sequencing rRNA, and to maximize the sequencing output from mRNA, a depletion of the rRNA was performed using the Ribo-Zero™ Magnetic Kit (Gram-Positive Bacteria) (Epicentre Biotechnologies). Four µg RNA was loaded into the Ribo-Zero™ kit and typical yields of mRNA were 3-5 % of the input amount. The success of the rRNA-depletion was checked with the Bioanalyzer. Forty ng of the resulting RNA was used as input for the ScriptSeq™ v2 RNA-Seq Library Preparation Kit (Epicentre Biotechnologies) to produce libraries compatible with the Illumina MiSeq. The kit fragments the RNA, then synthesizes cDNA, and finally incorporates an optional index sequence. Four different indices were incorporated into the libraries allowing four samples to be sequenced simultaneously on the MiSeq. Typically, this produced between 3 and 5 million reads per sample. Before sequencing, the libraries were analyzed using the Bioanalyzer High Sensitivity DNA chip (Agilent) to assess the quality and amount of library DNA produced. The libraries were pooled and diluted according to the MiSeq library preparation manual and sequenced using the MiSeq Reagent Kit v2 50 bp. The MiSeq was instructed to sequence 1x50 bp and to only output FASTQ-files with the reads. The RNAseq reads from the MiSeq have been deposited to Genbank under BioProject accession number PRJNA217316.

The reads were aligned to the annotated coding regions of the Cow1 isolate by using Bowtie 2 with the ‘very-sensitive’ setting.(5) Bowtie 2 was set to align locally and to output information only on successfully aligned reads. The SAM output file was converted to its binary BAM-version and then sorted using the “view -bS” and “sort” functions of SAMtools v. 0.1.18.(6) The sorted BAM-file was used as input for Cufflinks which assembles the aligned RNA-Seq reads into transcripts.(7) Following cufflinks, cuffmerge and cuffdiff 2 were used to merge the transcripts from two samples into one. Cuffdiff 2 compares the levels of expression for the samples and tests them for significance. The quartile normalization function, available both in cufflinks and cuffdiff 2, was not used.

Determining the *in vitro* mutation rate

The Cow2-isolate was cultivated on a horse-blood agar plate overnight at 37°C after which a single colony was spread onto three new plates with an inoculation loop. After another overnight incubation at 37°C, a single colony from each plate was spread onto a new plate. This process was repeated 9 times for at total of 10 passages.

A single colony was used to inoculate 5 ml of Luria-Bertani-medium from the starting plate and from each of the three plates after passages 5 and 10. The cultures were then extracted for DNA and the DNA sequenced on the Illumina MiSeq; mutations were determined as described under 'DNA-sequencing and mutation discovery'. The mutation rate was estimated by assuming a probability of 0.333 (one out of three) that the bacteria had not acquired a mutation after 10 passages. Thus, if the probability of acquiring a mutation in each passage is x, the probability of not acquiring a mutation is (1-x) and for all 10 passages $(1-x)^{10}$. Thus, $x=1-0.333^{1/10}$. A colony was estimated to contain 8 million bacteria, corresponding to 23 division cycles of exponential growth.

Quantifying the intra-animal mutation heterogeneity with amplicon NGS

Primers were designed to amplify the regions containing the determined SNPs/indels. PCR was performed using these primers and samples used were the original DNA-extractions from the samples analyzed during the outbreak. They consisted of around 40 µl of elute from the original DNA-extraction isolated with the Qiagen EZ1 Advanced kit (Qiagen, Hilden, Germany) and stored at -20°C. Samples were used together with the primers for the mutation that the DNA-sequencing had shown to exist in that sample. Different samples from the same animal were also analyzed for all mutations found in isolates from that animal.

The total reaction volume was 25 µl and consisted of 12.5 µl PerfeCTa® Multiplex qPCR SuperMix (Quanta BioSciences, Gaithersburg, MD, USA), 200 nM of each primer, and 2 µl of undiluted sample. The PCRs were performed on the 7500 Fast Real-Time PCR System (Life Technologies) using the following temperature profile: 95°C for 3 minutes for initial denaturation, then 35 cycles of 95°C for 10 s, and 60°C for 60 s. Agarose-gel analysis showed that only one product per PCR had formed. The concentration of the amplicons was determined using the Qubit® dsDNA BR Assay Kit (Life Technologies). To prepare the amplicons for sequencing, the standard protocol for the Nextera XT kit (Illumina) was followed except for the bead-based normalization part. This part was replaced by first measuring the created libraries on the Bioanalyzer and the High Sensitivity DNA chip (Agilent), then diluting and pooling the samples according to the instructions for the MiSeq (Illumina). Sequencing was performed on the MiSeq (Illumina) with 2x250 bp reads. The reads were aligned to FASTA-files of the regions of the mutations using the Bowtie2 aligner.(5) Around 98-99 % of the reads were successfully aligned to each ~2 kb area, amounting to between 100,000 and 400,000 reads. The ratio of a certain mutation was calculated by counting the number of reads supporting the two variants. The noise levels were between 0.01 - 0.5 % and were estimated by counting other possible mutations in the area of each sought mutation.

References

1. Gordon D, Abajian C, & Green P (1998) Consed: a graphical tool for sequence finishing. *Genome Res* 8(3):195-202.
2. Delcher AL, Bratke KA, Powers EC, & Salzberg SL (2007) Identifying bacterial genes and endosymbiont DNA with Glimmer. *Bioinformatics* 23(6):673-679.
3. Kurtz S*, et al.* (2004) Versatile and open software for comparing large genomes. *Genome Biol* 5(2):R12.
4. Wielinga PR*, et al.* (2011) A multiplex real-time PCR for identifying and differentiating *B. anthracis* virulent types. *Int J Food Microbiol* 145 Suppl 1:S137-144.
5. Langmead B & Salzberg SL (2012) Fast gapped-read alignment with Bowtie 2. *Nat Methods* 9(4):357-359.
6. Li H*, et al.* (2009) The Sequence Alignment/Map format and SAMtools. *Bioinformatics* 25(16):2078-2079.
7. Trapnell C*, et al.* (2010) Transcript assembly and quantification by RNA-Seq reveals unannotated transcripts and isoform switching during cell differentiation. *Nat Biotechnol* 28(5):511-515.
